# Endogenous testosterone and exogenous oxytocin influence the response to baby schema in the female brain

**DOI:** 10.1038/s41598-018-26020-4

**Published:** 2018-05-16

**Authors:** Sarah K. C. Holtfrerich, Roland Pfister, Alexander T. El Gammal, Eugen Bellon, Esther K. Diekhof

**Affiliations:** 1 Universität Hamburg, Faculty of Mathematics, Informatics and Natural Sciences, Department of Biology, Institute of Zoology, Neuroendocrinology Unit, Martin-Luther-King-Platz 3, D-20146 Hamburg, Germany; 2 Department of Psychology, Julius-Maximilians-University of Würzburg, D-97070 Würzburg, Germany; 3 General, Visceral and Thoracic Surgery Department, University Medical Center Hamburg-Eppendorf, D-20246 Hamburg, Germany

## Abstract

Nurturing behavior may be critically influenced by the interplay of different hormones. The neuropeptide oxytocin is known to promote maternal behavior and its reduction has been associated with postpartum depression risk and child neglect. Contrariwise, the observed decrease in testosterone level during early parenthood may benefit caretaking behavior, whereas increased testosterone may reduce attention to infants. Here we used functional magnetic resonance imaging to investigate the interactive influence of testosterone and oxytocin on selective attention to and neural processing of the baby schema (BS). 57 nulliparous women performed a target detection task with human faces with varying degree of BS following double-blinded placebo-controlled oxytocin administration in a between-subjects design. Our results support the idea that oxytocin enhances attention to the BS. Oxytocin had a positive effect on activation of the inferior frontal junction during identification of infant targets with a high degree of BS that were presented among adult distractors. Further, activation of the putamen was positively correlated with selective attention to the BS, but only in women with high endogenous testosterone who received oxytocin. These findings provide initial evidence for the neural mechanism by which oxytocin may counteract the negative effects of testosterone in the modulation of nurturing behavior.

## Introduction

The survival of the offspring is crucial for one’s reproductive success. Newborns may therefore carry specific features that act as key stimulus, which automatically captures attention and motivates actions such as caretaking. The baby schema (BS) is such a key stimulus. In the infant face it is defined as a combination of child characteristic features like big eyes, small nose, chubby cheeks, and higher forehead^1^. Humans bear high costs in raising their offspring, because pregnancy and production of maternal milk are energetically expensive^2^, which should make newborns highly relevant, at least for mothers. But since humans practice alloparental care^3^ and the BS is further known to be a universally relevant stimulus^4^ the BS should be highly significant for women in general. The increased cuteness of infant faces in comparison to adults may therefore prioritize selective attention^5,6^ and may enhance evaluation of these stimuli as socially relevant for actions like caretaking^7^.

There is already evidence for an adaptive neural mechanism through which the perception of cuteness as a feature of the BS is most likely promoted in humans. Accordingly, cute baby faces have repeatedly yielded activations of regions of the mesolimbic reward system^8,9^. For example, Glocker *et al*.^10^ observed an increase of activation in the nucleus accumbens in women while evaluating pictures of babies with increasing BS (i.e., low BS < unmanipulated BS < high BS). Interestingly, the mesolimbic reward system may also be highly responsive to the influence of two hormones that have opposing roles in human nurturing behaviors. Firstly, a high oxytocin (OT) receptor density can be found in the mesolimbic reward system of social mammals^11^ and increased activation of the ventral tegmental area to images of crying infants after OT treatment suggested that OT influences the reward value of stimuli carrying the BS^12^. Further, OT administration increased the preference for pictures of young children. This preference was determined through rs53576G homozygote participants (a polymorphism in the OT receptor gene)^13^. Finally, OT has been consistently associated with higher maternal attachment and increased caretaking in humans^14,15^. The androgen testosterone (T) may also have reinforcing effects in the mesolimbic reward system^16^. Yet, in contrast to OT it may compromise behaviors associated with maternal care (e.g. nurturing)^17^. Previous studies have noted a reduced T level during early parenthood and this decline appeared to be associated with the quality of childcare^18–22^ (but see^23^).

However, the interactive influence of T and OT on neural processing of the BS currently remains elusive. Only a few behavioral studies have so far assessed their combined influence on aspects of nurturing behaviors. In the context of paternal caretaking behavior Weisman and colleagues^24^ found that OT administration led to an unexpected short-term alteration of endogenous T, whereby nurturing behaviors like social gaze also increased. Given that women are the main caretakers during the first months after childbirth when infants depend on maternal milk, and yet sometimes show impairments like postpartum depression or child neglect^25^, it is of crucial importance to take a closer look on these interactive influences in women as well. In nulliparous women a higher endogenous T reduced selective attention to infant targets in the context of adult distractors^6^. Importantly, attention to infant stimuli increased after OT administration, but only in women with high endogenous T. This suggests that OT may oppose the negative effects of T on nurturing behavior in women^6^. There are already some indications on the behavioral level that T and OT may have gender-specific impacts on parental behavior. Gordon and colleagues^26^ found that high T levels in fathers negatively influenced the association between OT and paternal behavior, whereas in mothers high T levels evoked positive associations. Nevertheless, the neural mechanisms underlying the effect of the OT by T interaction on selective attention to infants in general and the highly relevant key stimulus BS in particular remains elusive.

In order to further our understanding of the nature of hormonal interactions in nurturing behavior, the present study used functional magnetic resonance imaging (fMRI) and a double-blind placebo-controlled OT administration between-subjects protocol to examine the influence of exogenous OT and endogenous T on selective attention to the BS in nulliparous women. To assess selective attention to the BS we used an implicit association task. Implicit association tasks have the advantage that the degree of attentional capture by an infant as opposed to an adult face can be determined implicitly by the mean reaction time (RT) of the participant, with a shorter RT indicating faster attentional processing of the baby schema and increased action. Previous neuroimaging studies combined passive viewing and explicit evaluation tasks (e.g., cuteness perception on a Likert-Scale), which are more vulnerable to experimenter demand effect. Further, these studies do not allow the assessment of the mechanism that promotes faster reactions to infants. Building on previous findings^6^ we hypothesized that OT administration would compensate the negative effects of high endogenous T on attentional processing of the BS, probably through the modulation of activation in key nodes of the mesolimbic reward system.

## Results

### Behavioral data

The influence of the endogenous T concentration (measured out of one saliva sample that was collected directly before administration) on attention to babies was analyzed with a 3 (morph type: low BS, natural BS and high BS) × 2 (treatment: OT vs. placebo) × 2 (T: median split of the sample that was collected directly before administration) ANOVA. Of particular interest was the significant three-way interaction between the factors “*morph type*”, “*treatment*” and “*T*” (*F*_2,104_ = 4.359; *p* = 0.015; see Table S1 for details). Building on previous research^6^ we assumed that women with high endogenous T derive greater benefit from OT treatment than women with low endogenous T and hence will become more sensitive for the BS. We found that OT-treated women with high endogenous T exhibited an attentional preference for the natural BS (positive Delta-RT: *M*_highT_ = 6.4 ± SEM 24.2 ms) compared to OT-treated women with low endogenous T (positive Delta-RT: M_lowT_ = −50.6 ± SEM 16 ms) but these results remained on statistical trend level [Bonferroni-corrected statistical threshold was p ≤ 0.0166 (*t*_27_ = 1.86, *p* = 0.03; one-tailed, a priori hypothesis based on^6^)]. In the placebo group women with low endogenous T directed less attention to the plus morphed babies (*M*_lowT_ = −39.1 ± SEM 21.3 ms) than women with high T concentrations (*M*_highT_ = 23.4 ± SEM 14.6 ms; *t*_25_ = 2.29, *p* = 0.015, one-tailed, a priori hypothesis based on^6^). The placebo-treated participants with high or low T did not show any differences in the preference for the natural BS.

Drug treatment did not influence the participants RTs to geometric figures. Yet, the type of target (F_2,110_ = 39.485; p < 0.001; n_p_^2^ = 0.418) and the interaction between target and distractor (F_2,110_ = 25.871; p < 0.001; n_p_^2^ = 0.32) influenced the participants’ RTs in the control task (see also Table S2). These results are consistent with the assumption that simple geometric forms could have emotional meanings and may even trigger an attentional bias^27^.

### Neuroimaging results

**Figure 1:**
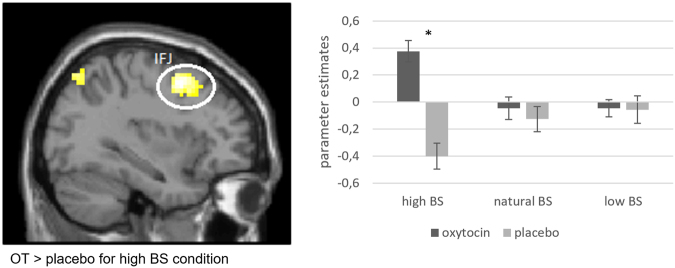
Increased activation of the inferior frontal junction (IFJ) for baby targets that were morphed towards higher BS > adult targets in the comparison of the OT and placebo group (MNI coordinates X, Y, Z [*t*-value]: −33, 5, 49 [4.62]). Activation is displayed in MNI space on an axial slice. Parameter estimates were extracted from spheres at the local maximum with a radius of 3 mm. They were significantly different between the OT and the placebo group for the high BS condition.

When contrasting “baby vs. adult target” for the different morph types, we found an effect of treatment in the direct comparison of the OT and placebo group in the left frontolateral cortex (MNI-coordinates [*t*-value; p_FWEcorr_] −33, 5, 49 [4.62; 0.023]), but only for the high BS condition (Fig. 1). The identified region was located at the intersection of the precentral sulcus and inferior frontal sulcus, which corresponds to the inferior frontal junction (IFJ)^28^. Additional t-tests on the parameter estimates extracted from the left IFJ, confirmed the difference in activation between the OT and the placebo group in the high BS condition (t_55_ = 4.43; *p* < 0.001) (Fig. 1). We could not find any difference in the direct comparison of OT > placebo in the unmanipulated or the low BS conditions.

**Figure 2:**
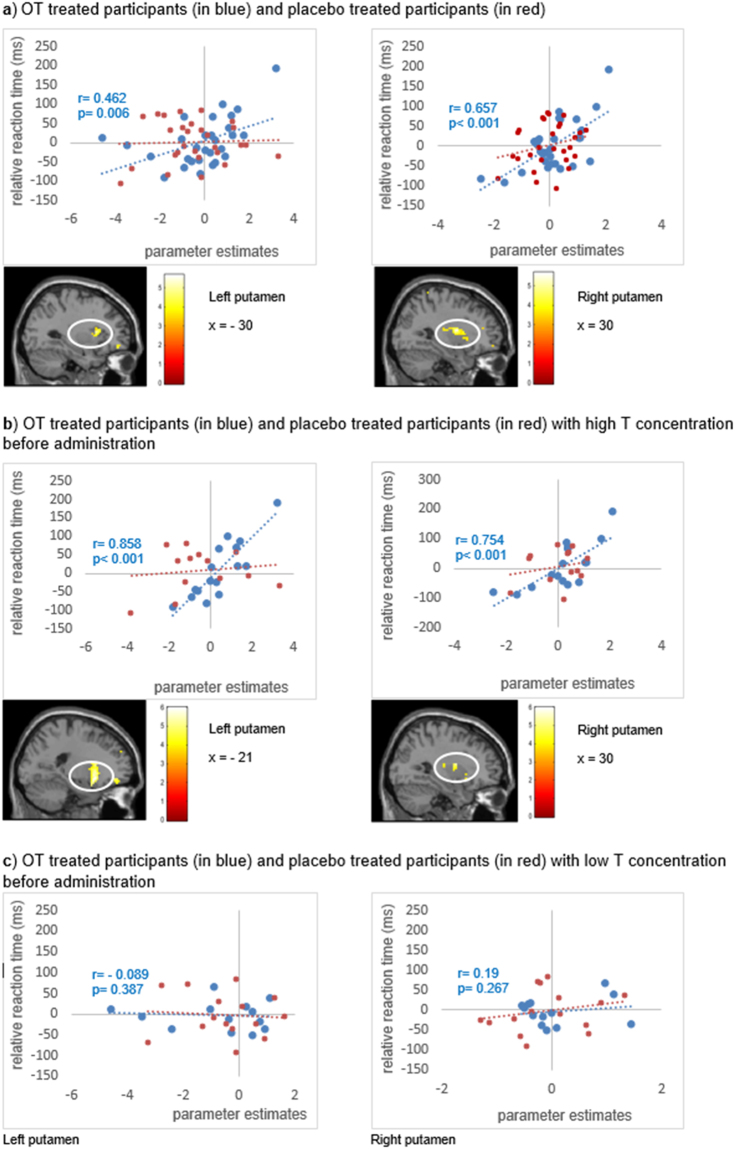
Degree of selective attention to babies for OT treated participants (in blue) or placebo treated participants (in red) with high or low T concentrations. (**a**) Activation in the left and the right putamen for baby relative to adult targets positively correlated with selective attention to babies after OT treatment (N = 29). Parameter estimates from local activation maxima (L:–21 14–11; R: 30–1 13; spheres with 6 mm radius) for the right and the left putamen are also displayed for illustration purposes. (**b**) Positive correlation of activation in the left and right putamen with selective attention to babies for OT treated women with high endogenous T concentrations (N = 16). (**c**) OT treated women with low endogenous T concentrations (N = 13) did not show activation of the putamen in relation to the relative RTs (i.e., selective attention to babies).

The SPM multiple regression analysis was used to analyze the influence of the degree of selective attention to babies on brain activation. For this, we used the mean Delta RT of all baby conditions from each subject and correlated them with the individual activation in the contrast of “baby vs. adult target”, separately for OT- and for placebo-treated participants. We found increased activation in the right putamen of OT-treated women (MNI-coordinates [*t*-value; p_FWEcorr_] 30, −1, 13 [5.65; 0.052]; Fig. 2a) that was positively related to selective attention to babies, but barely missed the FWE-corrected threshold in the whole-brain analysis. Yet, when extracting the parameter estimates from the right putamen, a significant positive correlation with selective attention to babies emerged (Fig. 2a). We also found a positive correlation with activation in the left putamen at the statistical threshold of *p* < 0.001, uncorrected (MNI-coordinates [*t*-value] −30, 14, 10 [4.85]; Fig. 2a). In the placebo group, two homologous clusters emerged in the posterior temporal cortex of which one survived the FWE correction on cluster level and one barely missed the statistical threshold (MNI-coordinates [*t*-value; p_FWEcorr_] 48, −22, −2 [5.69; 0.067]; MNI-coordinates [*t*-value; p_FWEcorr_] −66, −49, −2 [4.99; < 0.001]).

Following the behavioral results, we subdivided the sample in women with either low or high endogenous T concentrations. OT-treated women with high endogenous T showed significantly enhanced activation in the left and right putamen in the contrast of “baby vs. adult target” in association with the individual Delta-RTs as an indicator of enhanced selective attention to babies (i.e., a positive correlation). The positive correlation with the left putamen also surpassed the statistical criterion (MNI-coordinates [*t*-value; p_FWEcorr_] −21, 14, −11 [5.97; 0.005]; Fig. 2b), while the one with the right putamen was significant at p < 0.001, uncorrected (MNI-coordinates [*t*-value] 30, −1, 13 [5.33]; Fig. 2b). OT-treated participants with low endogenous T and participants of the placebo group did not show this positive correlation between activation in the putamen and selective attention to babies. The brain-behavior correlations based on the parameter estimates extracted from the left and the right putamen supported these results (Fig. 2c; Table S3).

## Discussion

The current study investigated the influence of exogenous OT and its interaction with endogenous T on brain activity during processing of infant faces, which varied in the intensity of BS. We found a positive effect of drug treatment in the direct comparison of OT versus placebo using the contrast of “baby versus adult target” in the IFJ, but only in the high BS condition. This finding supports the idea that OT may enhance attention to increased BS and may further promote one’s motivation to act. Additionally, we found a positive correlation between selective attention to babies, as indicated by increasing Delta-RTs, and increased activation in the left putamen following OT treatment. Interestingly, this correlation was specific for women with high endogenous T. Collectively, these findings may provide a hint to the neural mechanism by which OT may support the sensitivity for the BS and may counteract the negative effects of T in the modulation of nurturing behavior.

Based on our previous study^6^ we expected that OT administration would modulate attention to infant faces especially in women with high endogenous T. In line with this hypothesis, we found an interaction between the degree of BS, the treatment group and endogenous T level. OT-treated women with high endogenous T showed an attentional bias towards babies (positive Delta-RTs) compared to OT-treated women with low endogenous T (negative Delta-RTs), but only for the natural BS and this result remained on statistical trend level. We could not find further differences in the attention to babies between high and low endogenous T concentrations in the OT group, but the Delta RTs of the conditions with lower and higher BS were positive in both T groups. We suppose that adult pictures could also represent salient stimuli for nulliparous women in reproductive age, which may have rendered any behavioral effects rather small (see^8^ for review). Yet, the presence of a statistical trend in the OT-treated women with high endogenous T may conform to the idea that a system influenced by high levels of T may be more receptive to OT treatment, because in the female brain T could be directly converted into estradiol and thus modulate OT receptor density, which has been demonstrated in rats^29^.

We found a positive effect of treatment (OT > placebo) on processing of targets with higher BS in the left IFJ. The IFJ is located in the frontolateral cortex between the premotor and the prefrontal cortex and has been implicated in cognitive control^30,31^. Recent research further demonstrated its specific involvement in the control of selective visual attention^32^, action perception^33^ and the detection of behaviorally salient cues^34^. In addition, the IFJ has been associated with the understanding of action^35^, which may be considered as one basis of social cognition and empathy. Previous studies also detected increased activation during processing of infant faces in areas near our IFJ cluster. Caria and colleagues^36^ located activation in the precentral cortex (BA 6; MNI coordinates: 36–6 45) in the contrast of “unknown infant > adult faces”. A study on synchrony and specificity between the maternal and paternal brain found activation in the inferior frontal gyrus (IFG) for both sexes in response to own-infant-parent-interaction videos^37^. Finally, the IFG has also been related to OT- induced emotional empathy^38^. In our study, the activation of the IFJ was limited to babies with a high BS. Previous observations indicating that babies with stronger BS were treated preferentially^39^ and perceived as cuter^40,41^ provide some space for speculation, that the stronger BS could provoke an increased motivation to act. The findings of Glocker *et al*.^10^ who also found activation in the precentral gyrus (MNI coordinates: −42 0 29; −46 6 33; −51 8 36) across the three BS conditions, which was located near our cluster, could also provide indications for this speculation. Yet, it is difficult to distinguish between participants who searched for the target or avoided the distractors, which makes it difficult to interpret the difference between the OT group (positive parameter estimates) and the placebo group (negative parameter estimates). This would be of great interest and should be taken into account in a future study.

Additionally, we observed a positive correlation between activity in the left putamen and increased selective attention to babies in OT-treated women. The putamen belongs to the mesolimbic reward system and is part of the striatum. Equivalent to many other studies the detected activation appeared only in participants that were treated with OT^15^. We suppose that this finding supports the idea that OT increases the reward value of babies, probably as an adaptation to motivate caretaking behavior through mesolimbic-reward pathways^37^. This assessment is also supported by previous reports that have demonstrated OT-dependent activations in areas of the reward system as consequence of listening to crying babies (amygdala and insula^42^), to laughing babies (amygdala^43^) and processing of child pictures (globus pallidus^44^). Yet, the OT-related response in the putamen did not parametrically change with the amount of BS inherent to the presented pictures. This indicates a rather general mechanism that may ensure reflexive attention to babies regardless of their cuteness.

Even more importantly, the positive correlation between increased selective attention to babies and activation in the left putamen only remained significant in the high endogenous T group after OT administration. Only a few behavioral studies have so far examined the interactions between OT and T in women, and, to the best of our knowledge, our study is the first that used functional neuroimaging to assess the underlying neural mechanism. Here we used the T sample that was collected directly before administration to capture the current T state of the participant and not the habitual concentration, because we could not control for daytime fluctuations. This method also allows the comparability with previous research^26,45,46^. The single T sample collection before administration provided the opportunity to analyze the real-time T concentration at the measuring time. But, for the same reasons, we cannot exclude further T fluctuations that might have occurred during scanning, which may have weakened the stability of this single measurement and might therefore constitute a potential limitation of this study. However, the T concentration before administration and post-test concentration after measurement were highly correlated (r = 0.447; *p* < 0.001). This may suggest that T concentration may have been relatively stable during the actual scanning period.

The present study shows that the reward system may be involved in this attentional bias towards babies. Little is known about the precise interaction of OT and T in the brain. In the female brain the majority of T may be converted to estradiol via aromatase^47^, and estradiol can increase the number of OT receptors in the brain^29^. Yet, the observation of higher systemic T concentrations in women may indicate an impaired ability to convert it to estradiol, thus either leaving a higher amount of T in the circuitry that may counteract maternal behavior through an unknown mechanism or by leaving the number of OT receptors unaffected by reduced influence of estradiol. In a similar vein one may speculate that high OT concentrations may serve as a compensatory mechanism for high T concentrations, we presume that women with low T concentrations were less sensitive to OT administration, because low T concentrations should support nurturing behavior per se^6^. This would indeed explain why we could not find any activation in the brain reward system in women with low endogenous T after OT administration. But since babies nevertheless remain a strong social cue, this does not explain why neither in the low T nor in the placebo group the baby pictures were rewarding by themselves. We suppose that the young adult stimuli may also be socially salient for young participants, for instance as potential mates, and therefore could be equally rewarding and evoke similar activation in the brain reward system^48^. Our experimental design does not allow the isolation of the response to adult or infant faces, since pictures were always presented in combination, but only enables the identification of activational differences during processing of these stimuli. So we cannot answer this question. However, for reasons discussed before we presume that the brain of women with a high endogenous T concentration may be more sensitive to OT administration than women with a low endogenous T. One could therefore speculate that same age adult stimuli as well as infant pictures both constitute a strong social stimulus, but OT could function as an enhancer of reproductive behavior and direct attention towards infant stimuli in women with high T level, who may be more sensitive to OT. In this study, we focused on nulliparous women that reacted to unfamiliar infant or adult stimuli to investigate the neuroendocrine basics of the key stimulus in general. As oxytocin is especially known to influence attachment behavior^15^ it could be of great interest to use the same paradigm with familiar infants in further research. The results of this study are therefore limited to the general mechanism of the key stimulus BS and are not necessarily transferable to attachment behavior in mothers or other caretakers. Gordon *et al*.^26^ found a positive interaction between increased T levels and OT on maternal touch in mothers, while the effect in fathers was in the opposite direction. Their results may indicate that the interaction between T and OT may be sexually dimorphic. Therefore, our results on nulliparous women may resemble those in mothers but it is possible that they deviate to the results in fathers and men who have never sired a child. Although the findings of Gordon and colleagues^26^ were focused on parents, we assume that the results in mothers support our previous findings that OT seemed to promote adult-infant discrimination and increased attention to baby pictures, but only in women with high endogenous T^6^. OT is a peptide hormone that is critically involved in mother-infant attachment^49^. Especially lactating mothers show increased OT levels^50^. The artificial increase through intranasal administration could be closely related to the concentration found in new mothers (see Weisman *et al*.^51^ for the active drug effects reflected in saliva). Although there already exists excellent research about the active drug effects and reflection in saliva^49–51^, a direct comparison of mothers and non-mothers salivary OT concentration with and without OT administration would be of great interest. But yet, aside from oxytocin, postpartum hormonal changes have large transitions which makes the results of nulliparous women not necessarily comparable with mothers^52,53^.

The detected activations in the putamen correspond well with the literature and accentuate the previous research on oxytocin and maternal behavior^8,9,54^. Since we know very little about the interactions of T and OT in the female brain, we abstained from using an *a priori* region-of-interest approach and analyzed our data on whole brain level. However, findings in the IFJ are not common in the context of OT administration studies and in an *a priori* region-of-interest approach the IFJ would have probably not been included. This might limit the interpretation of this finding, although the activation of the IFJ survived the correction for multiple testing on a whole-brain basis. Future studies will therefore be necessary to show if this finding can be replicated in this context.

All of the participating women used hormonal contraceptives and were tested in the pill-phase. This was done to control for a potential pregnancy and to prevent cyclic changes in steroid hormone levels. But it’s important to note that previous research showed that the intake of oral contraceptives increased performance in affective responsiveness and that affective responsiveness was positively influenced by oral contraceptives (pill-intake phase versus pill-free week)^55^. Further, testosterone in females on oral contraceptives may be downregulated^56^. Therefore, using participants on hormonal contraceptives for an OT-intervention study may not represent the ideal model to detect OT sensitivity, that may be modulated by T. Yet, ethical concerns (increased pregnancy risk in women with a natural cycle), the fact that T level fluctuates across the natural cycle (e.g., rises during the first half), as well as our previous results in women on oral contraceptives^6^, led to our decision to test only women who received hormonal contraception in this between-subjects fMRI design. As a future direction, it would be of great interest to further examine normal cycling women and the influence of the intake of oral contraceptives on the recent results.

Collectively, the present findings support the idea of an adaptive hormonal mechanism that promotes selective attention to babies. Here, we demonstrate that an increased BS was associated with enhanced activation of the IFJ after OT treatment, a brain region implicated in cognitive control and the motivation to act. We also found that OT may modulate selective attention to babies through the recruitment of the reward system. We found a positive correlation between activity in the putamen and attention to baby faces. However, after accounting for endogenous T level, this correlation only remained significant in women with a high T concentration. These results are consistent with our previous findings^6^ and may indicate that OT possibly compensates high T concentrations and the reduced attention to infant stimuli through an enhancement of the reward value of babies.

## Material and Methods

### Participants

Sixty nulliparous female university students (mean age ± SD = 24.63 ± 3.08 years) participated in the study. All participants were right handed, healthy, and were not taking any medication except from hormonal contraceptives to prevent cyclic changes in T level. They also performed a pregnancy test on the test day and met the criteria to participate in an fMRI study.

Subjects provided written- informed consent and were paid for participation. The study was approved by the local ethics committee of the *Ärztekammer Hamburg*. All methods were performed in accordance with the relevant guidelines and regulations.

### Experimental Procedure and Paradigm

**Figure 3:**
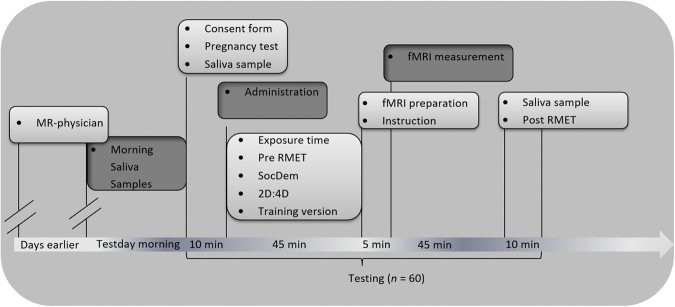
Time schedule of the experimental procedure. 60 participants were invited for testing. 57 participants could be included into the analysis (29 of the OT group; 28 of the placebo group). 20 of the participants performed the RMET test before the fMRI measurement and 37 participants performed the RMET test after the fMRI measurement.

Administration of OT was placebo-controlled and double-blind (see Fig. 3 for experimental schedule). Participants brought three saliva samples from home sampling and provided two further saliva samples at the test place (one before administration and one after measurement). In the scanner participants performed two versions of a target detection paradigm (see also Supplementary Material and Methods).

**Figure 4:**
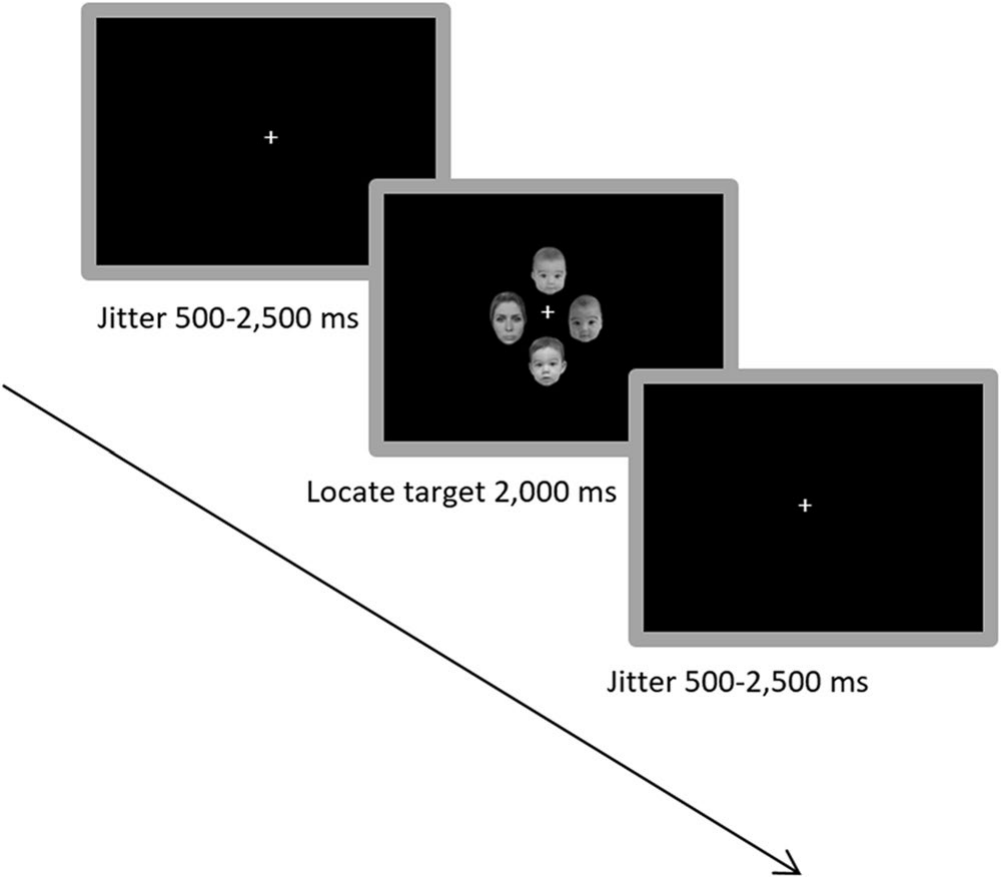
Illustration of the target detection paradigm with example images. The three picture of babies stand for the distractors *here* (photographs of either 3 adult faces or 3 infant faces were shown; the 3 infant faces had the same amount of BS [higher, unmanipulated or lower BS]). The adult picture on the left stand for target *here*, which could be either an adult or infant face of higher, unmanipulated or lower BS. The task was to identify the target that did not fit to the other pictures via button press.

The target detection paradigm was based on the odd-one-out principle (Fig. 4 for schematic illustration). In the first task subjects were asked to identify the odd-one-out on a display of four human faces, which was either an adult face (target) out of three infant faces (distractors) or an infant target out of three adult distractors, as fast and accurate as possible. The BS of the infant faces was parametrically manipulated (see also^6^ for the procedure). The same odd-one-out procedure was repeated in the baseline task, in which task geometric shapes (triangles, squares, or circles) were used (Supplementary Material and Methods for detailed information). The first paradigm was 16.4 minutes long and the baseline task was 6.4 minutes long.

### Hormone Samples

The salivary T samples were collected on the test day and analyzed in our in-house laboratory with a T luminescence immunoassay from IBL International (TECAN group global; Hamburg, Germany) (see Supplementary Material and Methods for detailed information).

### Hormone administration

OT was administered double-blind and placebo controlled in a between-subjects design. The participants self-administered 3 puffs in each nostril alternately of the unlabeled nasal spray. The amount of OT corresponded to 24 IU. The placebo spray consisted of chlorobutanol-hemihydrat (0.5%) with no active treatment. To ensure treatment efficiency^57^, exposure time was 45 min until the fMRI measurement began.

### Behavioral data analysis

57 participants were considered for analysis (29 were in the OT group and 28 were in the placebo group; for further details see Supplementary Material and Methods).

IBM SPSS statistics 19 was used to analyze the behavioral data. To represent selective attention to babies of different BS conditions, the relative reaction times (Deltas-RTs) were calculated by subtracting the low distracting condition (i.e., infant target and three adult distractors) from the high distracting condition (i.e., adult target and three baby faces as distractors) (see also^6^). For a subject with an increased sensitivity for the BS, we predicted higher Delta-RTs, resulting from the combined effect of increased distraction by infant faces in the adult target condition and more rapid selection of the infant target in the first condition.

Following the procedures used previously^26,46,58^,our analysis of T content focused on the one saliva sample that was collected directly before treatment. Based on the endogenous T concentration, we calculated the median to separate the participants in two groups of either high or low endogenous T concentration (see^6^ for a similar procedure).

We used a repeated measures ANOVA and post hoc *t*-tests to assess the effects of the factors ‘*morph type*’ (low BS, unmanipulated BS, high BS), ’*treatment group*’ (OT or placebo) and ‘*endogenous T concentration*’ (high or low T) on the Delta-RTs. Bonferroni correction yielded a corrected statistical threshold of p ≤ 0.0166 and a corresponding statistical trend level of p ≤ 0.033.

For analysis of the baseline task with geometrical shapes we performed a repeated measures ANOVA with the factors ‘*target figure’* (triangles, squares, or circles), ‘*distractor figures’* (triangles, squares, or circles) and ‘*treatment group’* (OT or placebo) on the RTs.

Statistical effects are considered significant at p < 0.05 (two-tailed), if not otherwise indicated. If the sphericity assumption was not met in the ANOVA, we report the Greenhouse-Geisser corrected values. Since all data followed the assumed normal distribution, we used post-hoc *t-tests*.

### fMRI data analysis

Participants performed the paradigm in a 3 Tesla Siemens fMRI Scanner at the University Medical Center Hamburg-Eppendorf (see Supplementary Material and Methods for details).

On the first level, we used a general linear model (GLM) for statistical analysis of event-related activity. A vector with the temporal onsets of the experimental conditions was convolved with a canonical hemodynamic response function (hrf) to produce the predicted hemodynamic response to each experimental condition. The conditions ‘*target*’ (adult or infant), ‘*morph type*’ of the baby (higher, unmanipulated or lower BS), ‘*gender*’ (female or male) and the baseline task with the geometric figures were modeled as regressors. Linear t-contrasts were defined for assessing the specific effects of the varying amount of BS.

On the second level, a random effects analysis was performed. For this we used a full factorial 3 by 2 repeated-measures analysis of variance (ANOVA) that included the within-subject factor ‘*morph type*’ (3 steps) and the between-subject factor ‘*treatment group*’ (2 steps). *T*-tests tested for specific differences between conditions.

We also assessed whole-brain correlations for analysis of the association between treatment-related activations and selective attention to babies (Delta RTs). For this purpose we used the multiple regression routine implemented in SPM8. For the display of these behavior-brain correlations individual parameter estimates were extracted with marsbar-0.44.

A whole-brain correction for multiple testing using the family-wise error (FWE) on the cluster level was applied to all analyses, if not indicated otherwise. For display purposes we used a threshold of p < 0.001, uncorrected.

### Data availability

All data analyzed and generated during this study are available from the corresponding author on reasonable request.

## Electronic supplementary material

Supplementary Material
