# Supplementary material for: Endogenous testosterone and exogenous oxytocin influence the response to baby schema in the female brain
Source: Sci Rep. 2018 May 16;8:7672. doi: 10.1038/s41598-018-26020-4 (PMC5955922; doi:10.1038/s41598-018-26020-4)
Supplement: Supplementary file 1 — Supplementary Material [file 41598_2018_26020_MOESM1_ESM.pdf]

Supplementary Material

for

Endogenous testosterone and exogenous oxytocin influence the response to baby schema in the female brain

Sarah K. C. Holtfrerich [1] *, Roland Pfister [2], Alexander T. El Gammal [3], Eugen Bellon [3], Esther K. Diekhof [1]

[1] Universität Hamburg, Faculty of Mathematics, Informatics and Natural Sciences, Department of Biology, Institute of Zoology, Neuroendocrinology Unit, Martin-Luther-King-Platz 3, D-20146 Hamburg, Germany

[2] Department of Psychology, Julius Maximilians University of Würzburg, D-97070 Würzburg, Germany

[3] General, Visceral and Thoracic Surgery Department, University Medical Center Hamburg-Eppendorf, D-20246 Hamburg, Germany

1. *Corresponding author: Sarah K. C. Holtfrerich, Universität Hamburg, Faculty of Mathematics, Informatics and Natural Sciences, Department of Biology, Institute of Zoology, Neuroendocrinology Unit, Martin-Luther-King-Platz 3, D-20146 Hamburg, Germany, sarah.holtfrerich@uni-hamburg.de, Tel.; +49-40-428389213, Fax: +49-40-428389718

Supplementary Tables

Table S1: Results of repeated measures ANOVA.

ANOVA	***factor***	***F***	***df***	***error***	***p***	n_p^2
ANOVA with T						
	Morph	1.874	2	104	0.159	0.035
	Morph*treatment	2.606	2	104	0.079	0.048
	Morph*T	0.764	2	104	0.469	0.014
	Morph*treatment*T	4.359	2	104	0.015*	0.077
	Treatment	0.085	1	52	0.772	0.002
	T	1.135	1	52	0.292	0.021
	Treatment*T	0.042	1	52	0.838	0.001

Significant effects at $p<0.05$ are marked with *

Table S2: Post hoc t-tests of the baseline task (*n* = 57). In this task geometric figures were used as stimuli (triangles, squares, or circles).

Paired t-tests	*target*	*distractor*	*mean RT (ms)*	*SEM*	*t*	*df*	*p*
pair 1	triangles	circles	825	19	-5.558	56	< 0.001
	triangles	squares	900	20			
pair 2	triangles	circles	825	19	2.109	56	0.039
	circles	triangles	799	16			
pair 3	triangles	squares	900	20	2.606	56	0.012
	squares	triangles	871	20			
pair 4	circles	triangles	799	16	1.052	56	0.297
	circles	squares	788	19			
pair 5	squares	triangles	871	20	4.280	56	< 0.001
	squares	circles	824	17			
pair 6	squares	circles	824	17	3.461	56	0.001
	circles	squares	788	19			

Table S3: Results of significant whole-brain activation in OT-treated women when processing babies relative to adult targets in general and separated by endogenous T.

	Oxytocin group (n= 29) *Positive correlation with attention to babies (Delta-RT across all morph types)*		***Oxytocin group with high T (n= 16)*** *Positive correlation with attention to babies (Delta-RT across all morph types)*		***Oxytocin group with low T (n= 13)*** *Positive correlation with attention to babies (Delta-RT across all morph types)*	
Brain region	MNI-coordinates	t-value; cluster size	MNI-coordinates	t-value; cluster size	MNI-coordinates	t-value; cluster size
**L/R putamen**	30 -1 13	5.65[1]; 102	30 -1 13	5.33[2]; 34	-	-
	-30 14 10	4.85[2]; 49	-21 14 -11	5.97; 148	-	-

Activations are FWE-corrected at cluster level with a threshold of p<0.05, if not otherwise indicated.

[1] p<0.10, FWE-corrected

[2] p<0.001, uncorrected

Table S4: Mean values of the four different T measurements, age of the participants and empathy score for the OT and for the placebo group. None of the measured values differed between the OT and the placebo group (*oneway ANOVA*, $p<0.05$, two-tailed).

	**Oxytocin**			**Placebo**		
**Measurement**	**N**	**mean**	**SD**	**N**	**mean**	**SD**
Morning testosterone samples (pg/ml)	29	25.48	13.1	27	26.2	13.7
Proxy of prenatal testosterone (ratio of 2nd digit to 4th digit)	29	0.98	0.02	27	0.98	0.03
Testosterone sample before nasal spray (pg/ml)	29	25.02	20.9	28	18.74	9.9
Testosterone sample after fMRI measurement (pg/ml)	29	23.28	12.7	28	24.2	17.3
Age of participants	29	24.93	3.6	28	24.32	2.4
Empathy score (RMET)	29	26.1	3.5	28	25.07	4.9

Supplementary Material and Methods

Experimental Procedure and Paradigm

Consent forms, instructions, and Eppendorf tubes for saliva sampling were handed to the participant in advance. Participants were instructed to collect three samples of morning saliva on the day of measurement.

Measurement started with a clarification of open questions, followed by a pregnancy test of the participant. After a negative test the participants provided another saliva sample followed by the self-administration of the nasal spray (24 IE, three puffs per nostril). Administration was placebo-controlled and double-blind. After 45 min exposure time, fMRI measurement began [1]. During exposure time, participants performed a short training version of the paradigm (with pictures of adult and infant animal faces) and completed a social demographic questionnaire (SocDem). In order to ensure comparability of the groups, participants performed a reading the mind in the eye test (RMET – after [2]).Furthermore, we measured the 2nd to 4th digit ratio as a proxy of endogenous androgen reception [3]. For this purpose, we measured the length of the index and ring finger from the fingertip (most distal point) to the lowest fold of the finger (most proximal point) of the right hand with a common caliper (accuracy ± 0,02/ ± 0,001). Subsequently we divided the length of the index finger through the length of the ring finger (2nd:4th digit) (for similar procedure see [3])

In the scanner participants performed two versions of the target detection paradigm (for a similar procedure see [4])The first target detection paradigm had 240 trials with each condition shown 40 times (i.e. 3 adult conditions, with an adult target and three either higher, unmanipulated or lower BS faces as distractors, and 3 baby conditions, with a baby target of either higher, unmanipulated or lower BS and three adult faces of the same gender as distractors). Each trial lasted 2,000 ms. A fixation cross was shown between trials. Its duration was jittered for 500-2,500 ms and randomized between trials. The first paradigm was 16.4 minutes long.

The same odd-one-out procedure was repeated in the baseline task. In this task geometric figures were used as stimuli (triangles, squares, or circles). Each condition was shown 30 times (i.e., triangle target, square target, circle target with three distractors of the other shapes), so that the paradigm was 90 trials long. The baseline task was intended to control for the influence of the OT treatment on participants' RT in general. The baseline task was 6.4 minutes long.

Subjects were asked to identify the target (the one picture that didn't fit the other three pictures) as fast and as accurate as possible via button press. The button positions matched the picture positions. The time of trial appearance was not influenced by button press and hits or false responses were not displayed for the participant (see Figure 4 for schematic illustration of the target detection paradigm).

The fMRI measurement was followed by another saliva sample of the participant.

Hormone Samples

The salivary T concentrations of the participants were analyzed in our in-house laboratory. Eppendorf tubes (2 ml) and an instruction for saliva sampling were handed over in advance at the first meeting with the MR-physician. The participants were asked to collect three saliva samples over the course of 1 hour at home. They started directly after waking up on the day of testing. During the collection of morning saliva the participants were instructed to refrain from eating and consuming cigarettes or any products that could influence the hormone measurement (for example milk, chewing gum or lip balm). They could drink water between sampling intervals and were allowed to brush teeth directly after the first saliva sample, leaving a gap of at least 15 minutes between tooth brushing and the second saliva sample to avoid a potential blood contamination. One additional saliva sample was collected right before administration of OT or placebo and a final one was obtained after the fMRI measurement.

The samples were frozen at -20°C until analysis. For preparing an aliquot out of the three morning samples and to separate these and the other two samples from mucus, all samples were unfrozen and centrifuged at RCF 604 x g for 5 minutes (i.e., 3,000 rpm) in a common Eppendorf Minispin centrifuge. After further refreeze and defrost of the samples, they were analyzed with a T luminescence immunoassay from IBL International (TECAN group global; Hamburg, Germany). Each sample, seven standards and two controls were pipetted twice on the assay plate. The analysis was performed according to the IBL manual. Formal sensitivity of the assay kit lies between 1.8 pg/ml and 500 pg/ml T in saliva. The intra- and inter-assay coefficient variances were declared to range between 1.47 - 3.01 % and 4.04 – 6.96 %.

To control for a potential pregnancy and to prevent cyclic changes in T level, we exclusively examined participants that used hormonal contraceptives. But it’s important to note that previous research showed that the intake of oral contraceptives increased performance in affective responsiveness and that affective responsiveness was positively influenced by exogenous hormones through intake of oral contraceptives (pill-intake phase versus pill-free week [5]). Further, the intake of combined oral contraceptives may decrease T concentrations in women [6]. Therefore, it will be of great interest to examine the influence of the intake of oral contraceptives as opposed to a natural menstrual cycle in a future study.

Behavioral data analysis

Two participants had to be excluded because they missed more than 30% of the trials. Another participant felt unwell during the fMRI measurement and did not finish the task.

One participant of the placebo group failed to bring the morning saliva samples and another participant missed a fingertip on the ring finger of the right hand, so we could not measure her 2D:4D ratio. These participants were nevertheless included.

We performed one-way ANOVAs to evaluate the differences between OT treated participants versus placebo treated participants in the RMET score, digit ratio, age and T measurements. A potential T

modulation through OT administration was also analyzed with a one-way ANOVA. Mean T values ($T_{morning}$, T concentration before fMRI measurement, T concentration after fMRI measurement and digit ratio), age and reading the mind in the eye test score (RMET – after [2]) did not differ between the OT and the placebo group (see Table S4). The RMET score was not influenced by the OT treatment (M_{11} pre administration = 25.18 ± *SD* 4.35; M_{18} post administration = 26.66 ± *SD* 2.91; $F_{1,55}$ = 0.823; p = 0.368).

The T change (Delta of pre minus post measurement concentration) over the experiment did not differ in the OT or placebo group (OT: M_{DeltaT} = 1.74 ± *SD*; placebo: M_{DeltaT} = -5.45 ± *SD* 12.82; $F_{1,55}$ = 2.724; p = 0.105).

Based on these analyses, the treatment and the placebo group were considered as comparable.

For analysis of the interaction between treatment and T concentration we calculated the median of the T concentration out of the sample before nasal spray administration. The median of the T concentration was 17.96 ± 16.6 pg/ml (n = 57).

fMRI data analysis

The whole brain measurement was set to thirty-nine axial slices with a voxel size of 3 x 3 x 3 mm^3 (distance factor 25 %). A total of 696 image volumes were obtained parallel to the anterior commissure–posterior commissure plane adjusted in descending direction in two sessions (target detection paradigm – 498 image volumes; baseline task – 198 image volumes). The field of view was set to 216 mm. The interscan interval was 2 s and the echo time was 25 ms. Participants viewed the paradigm through a head-coil mounted mirror.
Imaging data were preprocessed and analyzed with SPM8 (Wellcome Department of Cognitive Neurology, University College London, London, UK). Coregistration, correction of movement-related artifacts (realignment and unwarping), corrections for slice-time acquisition differences and low-frequency fluctuations, normalization and spatial smoothing were included in preprocessing process.

Parameter estimates were extracted from spheres at the local maxima (*IFJ*: -33, 5, 49 with a radius of 3 mm and *putamen*: L: -21 14 -11; R: 30 -1 13; spheres with 6 mm radius).

References

1. Born, J. *et al.* Sniffing neuropeptides: a transnasal approach to the human brain. *Nat. Neurosci.* **5,** 514–516 (2002).

2. Baron-Cohen, S., Wheelwright, S., Hill, J., Raste, Y. & Plumb, I. The ‘Reading the Mind in the Eyes’ Test Revised Version: A Study with Normal Adults, and Adults with Asperger Syndrome

or High-functioning Autism. *J. Child Psychol. Psychiatry* **42,** 241–251 (2001).

3. van Honk, J. *et al.* Testosterone administration impairs cognitive empathy in women depending on second-to-fourth digit ratio. *Proc. Natl. Acad. Sci.* **108,** 3448–3452 (2011).

4. Holtfrerich, S. K. C., Schwarz, K. A., Sprenger, C., Reimers, L. & Diekhof, E. K. Endogenous testosterone and exogenous oxytocin modulate attentional processing of infant faces. *PLoS One* **11,** 1–19 (2016).

5. Radke, S. & Derntl, B. Affective responsiveness is influenced by intake of oral contraceptives. *Eur. Neuropsychopharmacol.* **26,** 1014–1019 (2016).

6. Zimmerman, Y., Eijkemans, M. J. C., Coelingh Bennink, H. J. T., Blankenstein, M. A. & Fauser, B. C. J. M. The effect of combined oral contraception on testosterone levels in healthy women: A systematic review and meta-analysis. *Hum. Reprod. Update* **20,** 76–105 (2014).
